# Floral symmetry and scaling relationships between tepal mass and area in the daylily (*Hemerocallis fulva*)

**DOI:** 10.3389/fpls.2025.1599033

**Published:** 2025-07-25

**Authors:** Hongyu Yan, Peijian Shi, Weihao Yao, Feixue Jiang, Long Chen, Linli Deng, Meng Lian, Yi Heng, Karl J. Niklas

**Affiliations:** ^1^ School of Integrated Circuits, Chongqing University of Posts and Telecommunications, Chongqing, China; ^2^ Bamboo Research Institute, Nanjing Forestry University, Nanjing, China; ^3^ College of Landscape Architecture, Nanjing Forestry University, Nanjing, China; ^4^ School of Integrative Plant Science, Cornell University, Ithaca, NY, United States

**Keywords:** bilateral symmetry, diminishing returns, plant serial, pollination organs, radial symmetry, tepals

## Abstract

Floral symmetry plays an important role in the evolution and ecology of flowering plants, yet quantifying the symmetry of the perianth remains challenging. Here, we quantify the floral symmetry of the daylily (*Hemerocallis fulva*) with a focus on tepal mass, area, and shape. *H. fulva* was selected for this study because its perianth exhibits weak bilateral symmetry, providing a unique opportunity to investigate floral forms that are transitional between radial and bilateral symmetry. Toward this end, the tepal fresh mass (FM), dry mass (DM), tepal area (*A*), and the ratio of tepal width to length (*W*/*L*) of 136 flowers of *H. fulva* were quantified. In addition, the tepal roundness index (RI) and the standardized index for bilateral asymmetry (SI) were calculated. For the purpose of comparison, the FM, DM, and *A* of 202 leaves were measured and calculated. Reduced major axis regression protocols were then used to fit the scaling relationships of mass vs. area for tepals and leaves. With the exception of *W*/*L*, there were no significant differences in the means of FM, DM, *A*, RI, and ln(SI) between any two of the three inner whorl tepals or between any two of the three outer whorl tepals. However, there were significant differences in the means of these six measures between inner and outer whorls of tepals. The 95% confidence intervals of the scaling exponents of FM vs. *A* and DM vs. *A* of the outer whorl included unity. In contrast, the lower bounds of the 95% confidence intervals of the scaling exponents of FM vs. *A* and DM vs. *A* of the inner whorl and leaves of *H. fulva* exceeded unity. Different metrics for size (i.e., tepal mass vs. area) and shape (i.e., the degree of deviation from a standard circle and the degree of bilateral symmetry) yield different assessments of *H. fulva* perianth morphometrics (i.e. radial vs. bilateral symmetry), thereby highlighting the challenge of assessing symmetry. The scaling relationships of perianth parts and leaves are statistically congruent and consistent with the phenomenon called “diminishing returns” and the classical hypothesis of serial homology.

## 1 Introduction

Symmetry plays an important role in physics, mathematics, and biology (Bahadur et al., 2019; Damerval et al., 2021) in part because it is generally sufficient to describe a variety of forms (Almeida and Galego, 2005; Manuel, 2009; Yu et al., 2022). In biology, symmetry also reflects how organisms adapt three-dimensionally to their environment (Damerval et al., 2021; Yu et al., 2022). This is particularly true for angiosperms whose floral symmetries affect pollination and thus seed production and fitness (Citerne et al., 2010; Jiang and Moubayidin, 2022). A typical flower consists of several organ-types (i.e., sepals, petals, stamens, and carpels), with each organ-type fulfilling a specific function (Jiang and Moubayidin, 2022). The ground plan consisting of a short axis with ovules protected by carpels, followed by stamens, and the perianth, which is typically composed of petals and sepals, is arranged sequentially from the center outward (Bateman et al., 2006; Reyes et al., 2016; Damerval et al., 2021). Thus, the flower has a well-conserved ground plan that can nevertheless manifest many different adaptive phenotypes (Citerne et al., 2010). Among these phenotypic variations, floral symmetry plays an important role because it can influence plant-pollinator interactions (Regal, 1982; Endress, 2001; Sargent, 2004; Wang et al., 2015; Vujić et al., 2015; Carleial et al., 2017; Savriama, 2018; Spencer and Kim, 2018; Wang et al., 2023).

Flowers are predominantly symmetrical and rarely asymmetrical (Citerne et al., 2010; Endress, 2012). Among symmetrical flowers, there are two main types of symmetry: actinomorphy (radial symmetry) and zygomorphy (bilateral symmetry) (Endress, 1999; Citerne et al., 2010; Endress, 2012; Jiang and Moubayidin, 2022; Naghiloo, 2020). Radially symmetric flowers are divided into equal halves by three or more planes of symmetry (Naghiloo, 2020; Jiang and Moubayidin, 2022), whereas bilaterally symmetric flowers are divided into two mirror images by a single plane (or axis) of symmetry (Naghiloo, 2020; Jiang and Moubayidin, 2022). In general, radially symmetric flowers are considered the ancestral state and are morphologically accessible to diverse pollinators from all directions (Soza et al., 2022; Wang et al., 2023). Bilaterally symmetric flowers are considered the derived state that can motivate pollinators with more precise pollen placement (Neal et al., 1998; Ushimaru et al., 2009; Ramírez et al., 2011; Soza et al., 2022). Although the transition from radial to bilateral symmetry may increase pollinator specificity (Fenster et al., 2009; Jesson and Barrett, 2002), it can also engender a greater reliance on specific pollinators, which presents a risk if reliable pollinators reduce in number and become extinct (Reyes et al., 2016; Wang et al., 2023). In addition, the symmetry of flowers is often correlated with their orientation. Most radially symmetric flowers are typically oriented horizontally, allowing pollinators to approach from multiple directions (Damerval et al., 2021; Jiang and Moubayidin, 2022), whereas bilaterally symmetrical flowers are usually oriented vertically, with only one direction displaying their unique symmetry (Ostler, 1976; Endress, 2001; Sargent, 2004; Citerne et al., 2010; Reyes et al., 2016; Wang et al., 2023).

Despite the importance of floral symmetry and its well-recognized role in the evolutionary dynamics of flowering plants, its quantitative analysis has presented a challenge, particularly because organic symmetry is often a size-dependent trait. One approach to this particular challenge is the use of scaling theory, which has revealed relationships among size-dependent traits such as mass and area (Niklas, 1994; Niklas et al., 2007). In this context, prior research has shown that the exponents governing the mass vs. area scaling relationships of foliage leaves typically exceed unity (i.e., increases in area typically fail to keep pace with increases in mass) a phenomenon known as “diminishing returns” (Niklas, 1994; Milla and Reich, 2007; Niklas et al., 2007; Wang et al., 2024). This phenomenon has important implications for the biomass allocation patterns of floral parts, which are considered homologs of foliage leaves (Wolff, 1774; Goethe, 1790; Eyde, 1975; Bailey, 2008; Guo et al., 2022).

**Figure 1:**
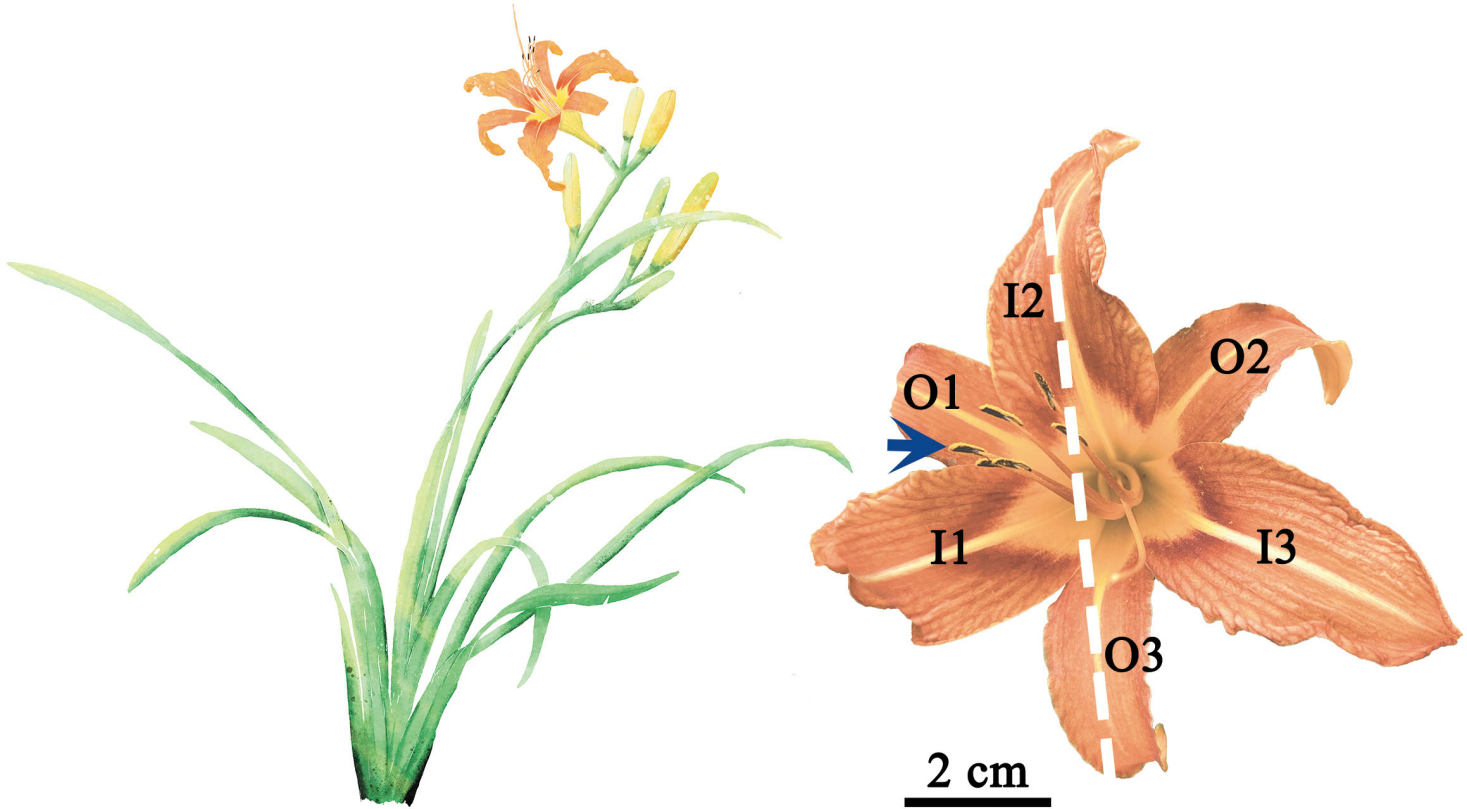
Representations of the above-ground morphology of a typical *H. fulva* plant (left) and a representative mature flower (right). I1, I2, I3, O1, O2, and O3 represent the tepals at the six positions denoted in the text and figures. The blue arrow denotes the stamens and stigma-styles; the white dashed line represents the reflection of insipient bilateral symmetry.

The goal of this study was to quantify the floral symmetry of the daylily (*Hemerocallis fulva*) and to explore the scaling relationships of tepal mass vs. area and leaf mass vs. area. *H. fulva* (Asphodelaceae) was selected for this study not because it exhibits strict bilateral symmetry, but because it represents a transitional floral form. The flower’s slight bilateral symmetry, as shown by the curvature of its stamens and stigma (**Figure 1**), allows us to examine how different metrics may converge or diverge in assessing floral symmetry. Studying such a transitional species helps elucidate the complexity of symmetry as a continuous trait, rather than a binary state, and highlights the challenges in quantifying it rigorously. *H. fulva* is broadly available in temperate and subtropical regions of China, Israel, Afghanistan, and Southeast Asia (Chen and Junko, 2000). Its flower (known as “Jin Zhen Cai” in China) has been used as a “vegetable” and medicinal herb for 3,000 years (Hsu et al., 2023; Lei et al., 2024).

## 2 Materials and methods

### 2.1 Species and collection of information

A total of 136 mature, undamaged flowers and 202 leaves of *H. fulva* were randomly collected at the Xinzhuang Campus of Nanjing Forestry University, Nanjing, Jiangsu Province, China (32°4′51ʺN, 118°48′57ʺE) in early June 2024. The samples were immediately placed in an insulated box and transported to the laboratory within 20 minutes. Each flower consists of six tepals, arranged in two whorls (inner and outer), each containing three tepals (**Figure 1**). Thus, a total of 816 tepals (136 flowers × 6 tepals) were collected and used in the study. The stamens and stigma of mature flowers of *H. fulva* consistently curve upwards (**Figure 1**), establishing a top, bottom, left, and right orientation for each flower. The outer and inner tepals (denoted as “O” and “I”) were labeled in a clockwise direction (1, 2, 3). Specifically, I2 and O3 correspond to the top and bottom, I1 and O1 to the left, and O2 and I3 to the right (**Figure 1**).

### 2.2 Image processing and data acquisition

The fresh and dry mass (FM and DM, respectively) of tepals and leaves were measured because the former reflects the mass that must be mechanically supported, whereas dry mass is a measure of carbon allocation, with each metric providing different functional traits (Kramer and Boyer, 1995; Niklas and Spatz, 2012), using an electronic scale with a precision of 0.01 g (JM-A3002; Chaozeheng Equipment Company Limited, Zhuji, Zhejiang, China). Each tepal and leaf was subsequently scanned at 600-dpi resolution with an Epson photo scanner (V550, Epson Indonesia, Batam, Indonesia). Adobe Photoshop 2021 (version 22.4.2; Adobe Systems Incorporated, San Jose, CA, USA) was used to obtain black and white images of tepal and leaf edges, which were saved as bitmap images at a resolution of 600-dpi. We used the Matlab (version ≥ 2009a; MathWorks, Natick, MA, USA) procedure developed by Shi et al. (2018a) and Su et al. (2019) to calculate the pixel values of each image and obtain the planar coordinates of tepal and leaf boundary points.

The “bilat” function in the “biogeom” package (version 1.4.3; Shi et al., 2024) based on the R software (version 4.2.0; R Core Team, 2022) was used to calculate the tepal area (*A*), tepal length (*L*, defined as the distance from the apex to the base of the tepal), tepal width (*W*, defined as the maximum distance between two points on a tepal profile through a straight line perpendicular to the tepal length axis), tepal perimeter, the standardized index for bilateral asymmetry (SI), and lamina area (LA). The ratio of tepal width to length (*W*/*L*) was also calculated.

Flower and leaf data are available in online **Supplementary Tables S1**, **S2**.

### 2.3 Tepal shape deviation from a standard circle and symmetry measures

The roundness index (RI) was used to measure the deviation of tepal or leaf shape from a standard circle (Niinemets, 1998; Peppe et al., 2011) and SI (Shi et al., 2018b; Mu et al., 2024) was used to assess the degree of bilateral symmetry.

**Figure 2:**
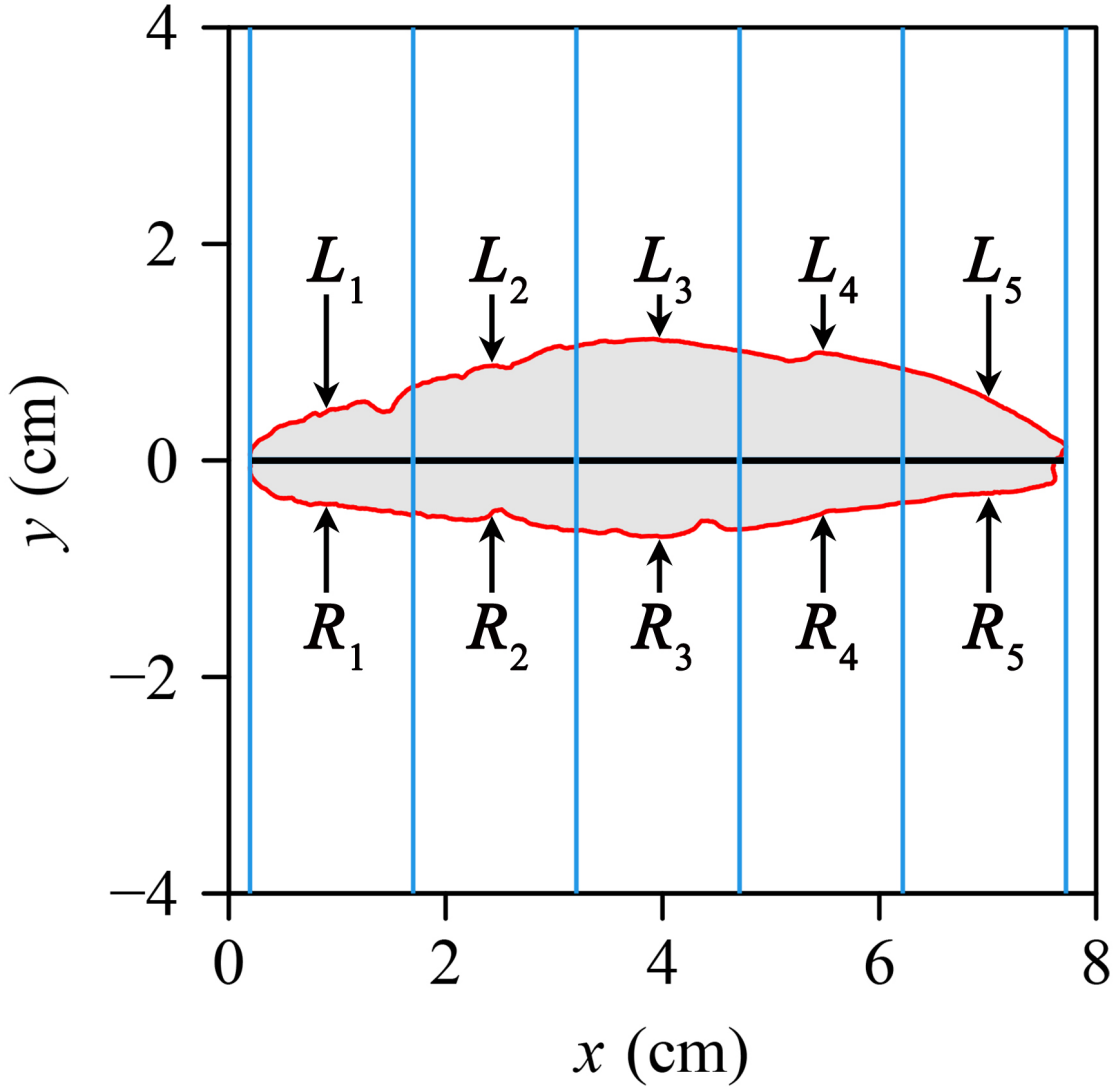
An illustration of the protocols used to calculate the standardized index of bilateral asymmetry (SI), showing only five equidistant strips as opposed to 1000 strips for simplicity, which were used in this study. The intersection between each strip and the tepal in each strip was divided into the upper (left) and lower (right) parts: *L*_1_ to *L*_5_ represent the areas of the left part, and *R*_1_ to *R*_5_ represent the areas of the right part.


RI was calculated using the equation


(1) $$\text{RI}=\frac{4\text{π}A}{P^{2}},$$

where *P* is tepal or leaf perimeter and *A* is tepal or leaf area. A larger RI reflects a smaller degree of shape deviation from a standard circle. To measure the extent of tepal bilateral symmetry, 1000 equidistant strips (rectangles) were established (**Figure 2**, where only five strips are displayed for clarity). According to Shi et al. (2018b), SI was developed to reduce the influence of organ size on symmetry measurement, and they used 999 strips in their analysis. Similarly, Mu et al. (2024) adopted 1000 strips for quantifying SI in leaves with complex shapes. While both studies did not systematically test the effect of strip number, their consistent use of a large number of strips suggests that such resolution improves stability. In this study, we adopted 1000 strips based on this precedent. The intersection between each strip was divided into upper (left) and lower (right) parts. The standardized index (SI) for tepal or leaf asymmetry quantifies the average of the relative area differences between the left and right parts for all the 1000 intersections of the strips. The mathematical expression of SI is

(2) $$\text{SI}=\frac{1}{1000}\sum_{i=1}^{1000}|\frac{L_{i}-R_{i}}{L_{i}+R_{i}}|,$$

where *i* represents the *i*-th strip, and *L_i_* and *R_i_* represent the left and right areas of the *i*-th strip, respectively. A smaller SI reflects a higher degree of bilateral symmetry; SI was log-transformed to ensure normality. Equations 1 and 2 were used to quantify the shape and bilateral symmetry of each tepal.

### 2.4 Data analysis

A power-law function was used to describe the scaling relationships of FM vs. *A* and DM vs. *A*, as well as LFM vs. LA and LDM vs. LA, i.e.,


(3) $$Y_{1}=\text{β}Y_{2}^{\text{α}},$$

where *Y*_1_ and *Y*_2_ are any two interdependent variables (e.g., tepal area and mass), β is the normalization constant, and α is the scaling exponent of the relationship between *Y*_1_ and *Y*_2_ (Niklas, 1994). When both sides of Equation 3 were log-transformed, the power-law function takes the form


(4) y=γ+αx,

where *y* = ln(*Y*_1_), *x* = ln(*Y*_2_), and γ = ln(β). The parameters γ and α in Equation 4 were determined using reduced major axis regression protocols (Niklas, 1994; Quinn and Keough, 2002). Tukey’s HSD test (α = 0.05) was used to determine differences in FM, DM, *A*, *W*/*L*, RI, and ln(SI). The bootstrap percentile method (Efron and Tibshirani, 1993; Sandhu et al., 2011) with 3000 bootstrap replicates was used to obtain the 95% confidence intervals (CIs) of scaling exponents of *Y*_1_ vs. *Y*_2_.

All calculations were performed and figures constructed using R software (version 4.2.0; R Core Team, 2022).

## 3 Results

**Figure 3:**
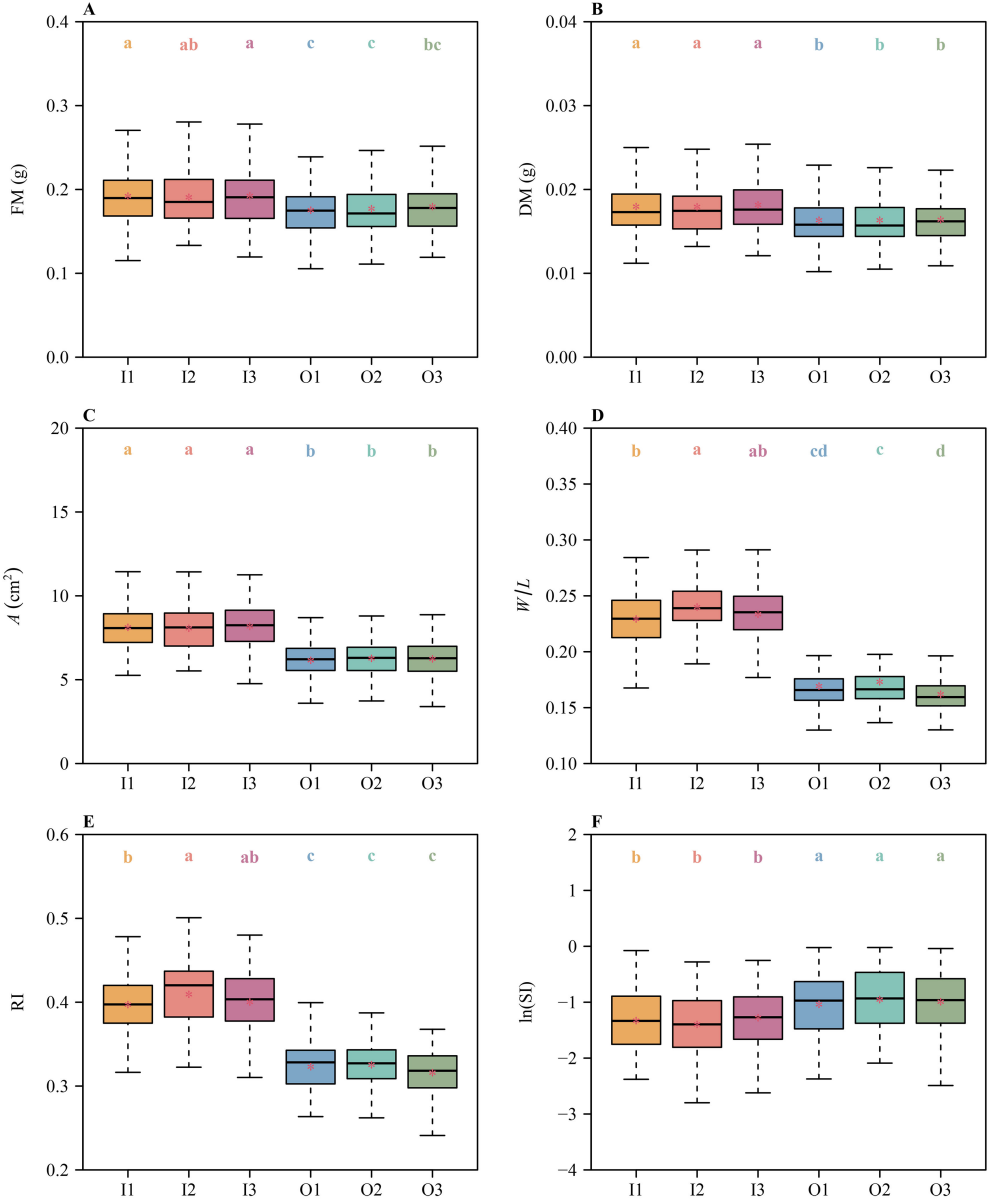
Boxplots of **(A)** tepal fresh mass, **(B)** tepal dry mass, **(C)** tepal area, **(D)** the ratio of width to length of tepals, **(E)** the tepal roundness index values, and **(F)** the natural logarithm of the standardized index for bilateral asymmetry values for each of the six tepals of *H*. *fulva*. The lowercase letters a–d indicate the significance of the difference in means between any two tepals based on the Tukey’s HSD test. Means with different letters are significantly different at *P* < 0.05. The horizontal solid lines represent the medians, and the asterisks within each box represent the means. In the *x*-axis label, I1, I2, I3, O1, O2, and O3 denote the six tepals (see **Figure 1**).

Different metrics for symmetry obtained different results. Specifically, there were no significant differences in the means of FM, DM, *A*, RI, and ln(SI) among I1, I2, and I3, or among O1, O2, and O3 (**Figure 3**). These metrics indicate that both the inner and outer tepal whorls manifest radial symmetry. In contrast, there were significant differences in the means of *W*/*L* between I1 and I2 and between O2 and O3 (**Figure 3D**), indicating that the inner and outer tepal whorls are not radially symmetrical.

**Figure 4:**
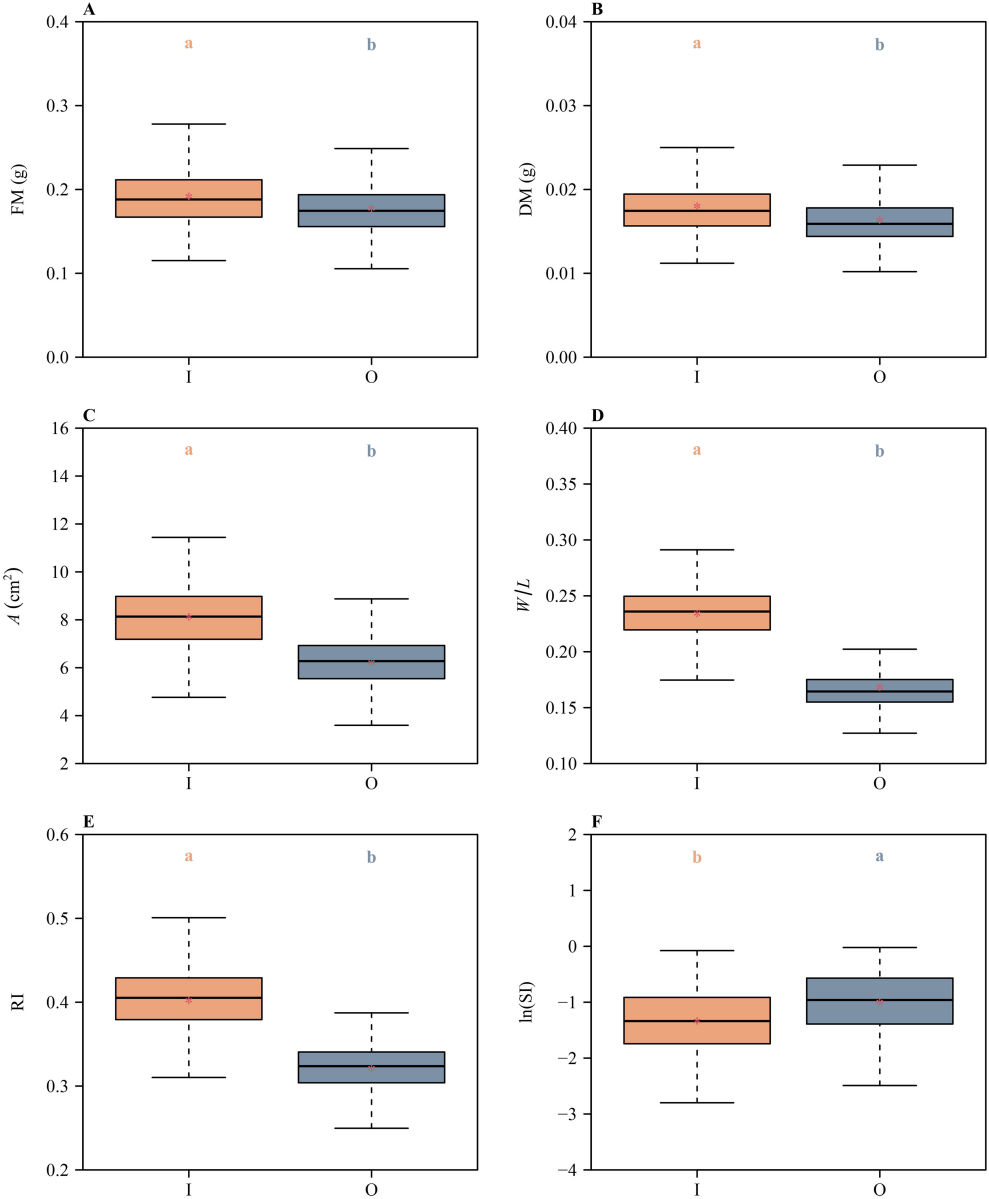
Boxplots of **(A)** tepal fresh mass, **(B)** tepal dry mass, **(C)** tepal area, **(D)** the ratio of width to length of tepals, **(E)** the tepal roundness index values, and **(F)** the natural logarithm of the standardized index for bilateral asymmetry values for the inner and outer tepal whorls of *H. fulva*. The lowercase letters a and b above the numerical values on the top of each box indicate the significance of the difference in means between any two tepals based on the Tukey’s HSD test. Means with different letters are significantly different at *P* < 0.05. The horizontal solid lines are the medians, and the asterisks within boxes represent the means. In the *x*-axis label, I and O represent the inner and outer whorl tepals, respectively.

Likewise, there were significant differences in the means of FM, DM, *A*, *W*/*L*, RI, and ln(SI) between the inner and outer whorls (**Figure 4**), indicating that the inner and outer whorls differed significantly in both size and shape, i.e., the inner whorl had larger means of FM, DM, and *A* compared to the outer whorl (**Figure 4**). These metrics indicated that the inner whorl is larger than outer whorl, reflecting a petal vs. sepal duality. In addition, the inner whorl had larger means of *W*/*L* and RI, and smaller means of ln(SI) than the outer whorl (**Figure 4**), i.e., the inner whorl manifested a broader shape, lower degree of shape deviation from a standard circle, and a greater degree of bilateral symmetry compared to the outer whorl.

**Figure 5:**
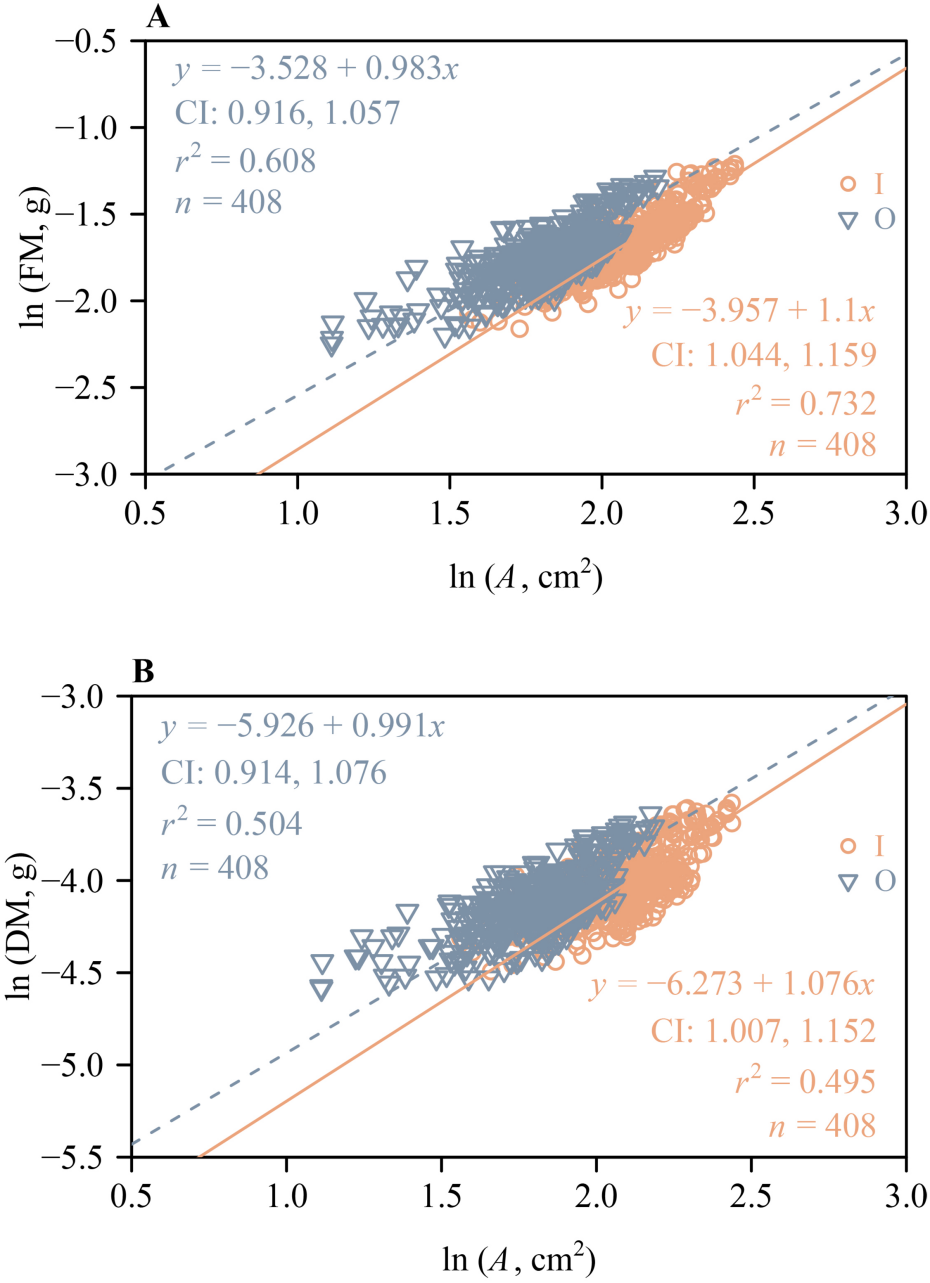
The log-log bivariate linear fit of tepal fresh mass vs. tepal area for the inner whorl and outer whorl of tepals of *H*. *fulva* **(A)**. The log-log bivariant linear fit of tepal dry mass vs. tepal area for the inner whorl and outer whorl tepals of *H*. *fulva* **(B)**. CI is the 95% confidence interval of the slope; *r*² is the coefficient of determination; *n* is the number of tepals for both inner and outer whorl tepals.

**Figure 6:**
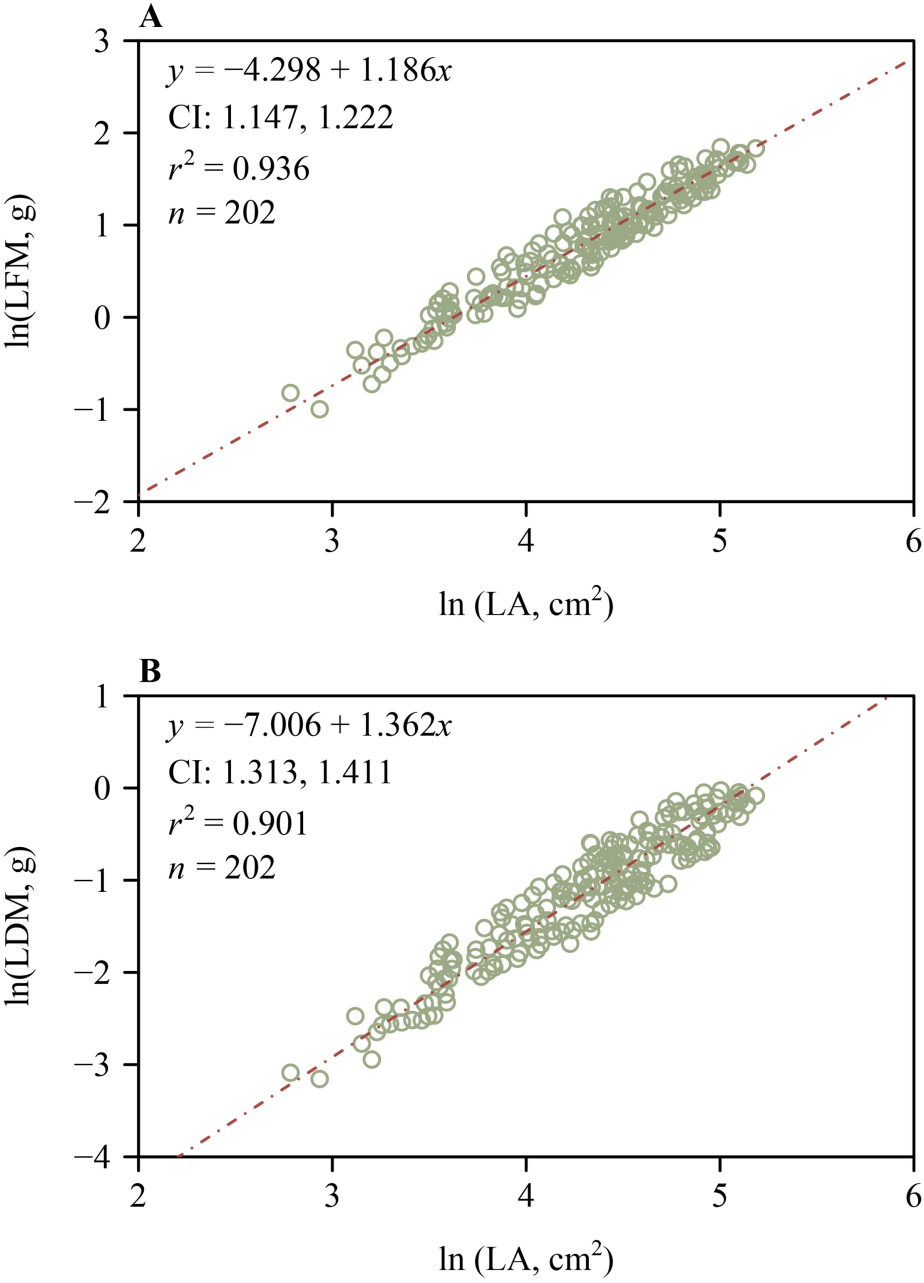
Log-log bivariate scaling relationships for leaf (lamina) *H. fulva*. **(A)** Lamina fresh mass vs. lamina area. **(B)** Lamina dry mass vs. lamina area. CI is the 95% confidence interval of the slope; *r*² is the coefficient of determination; *n* is the number of leaves in each sample.

The 95% CIs of the scaling exponents of both FM vs. *A* and DM vs. *A* for outer whorl included unity (**Figure 5**), indicating that both scaling relationships are isometric. However, the lower bounds of the 95% CIs of the scaling exponents of both FM vs. *A* and DM vs. *A* for the inner whorl exceeded unity (**Figure 5**), indicating a “diminishing returns” phenomenon, i.e., increases in tepal area fail to keep pace with increases in tepal mass. These scaling analyses were based on a total of 816 tepals, including 408 inner and 408 outer tepals (136 flowers × 3 tepals per whorl), with *n* = 408 representing the number of tepals in each whorl, as shown in **Figure 5**. Likewise, the lower bounds of the 95% CIs of the scaling exponents of LFM vs. LA and LDM vs. LA both exceeded unity (**Figure 6**), indicating “diminishing returns”, i.e., increases in lamina area fail to keep pace with increases in leaf mass.

## 4 Discussion

The data presented for the daylily *H. fulva* indicate that different metrics used to evaluate floral symmetry and size yield different results. The following sections discuss the implications of these results regarding the assessment of floral symmetry by comparing the inner and outer whorls of tepals, and the scaling relationships of lamina and tepal mass vs. area.

### 4.1 Floral symmetry

Floral symmetry has traditionally been described using the characteristics of perianth shape and size (e.g., symmetry, mass, and area) and the number and arrangement of stamens and carpels (Busch and Zachgo, 2009; Jiang and Moubayidin, 2022). The standardized index for bilateral asymmetry (SI) is a key metric for assessing the degree of bilateral symmetry (Shi et al., 2018b; Mu et al., 2024). However, we used it in combination with other size and shape metrics to capture distinct aspects of floral symmetry. No single metric can comprehensively describe the complex geometry of floral symmetry. By comparing multiple metrics, we aimed to provide a more comprehensive and nuanced evaluation of floral symmetry. Among these metrics, those quantifying shape have presented arguably the greatest challenges. For example, the data gathered for *H. fulva* indicate no significant differences in the means of fresh and dry mass (FM and DM) and the surface area (*A*) of the three tepals of the inner whorl or the three tepals of the outer whorl (**Figure 3**). Likewise, two metrics used to assess symmetry (RI and ln(SI)) revealed no differences among the three tepals for each of the two whorls. However, there are significant differences in the means of *W*/*L* between tepals I1 and I2, and between tepals O2 and O3 (**Figure 3D**). Thus, in terms of size, degree of shape deviation from a standard circle (RI), and the degree of bilateral symmetry (SI), both the inner and outer whorls of *H. fulva* are assessed as radially symmetrical, whereas in terms of *W*/*L*, they are asymmetric.

These seemingly conflicting results might be explained by the corrugated and often folded structure of *H. fulva* tepals (**Figure 1**). However, corrugations and folds do not easily explain differences in FM or DM, or in *W*/*L*, all of which are comparatively easily measured when tepals are weighed or flattened manually. A more likely explanation is rarely perfect in biology. Indeed symmetry is often “approximate”, with deviations emerging from finely tuned responses to microenvironmental conditions during development and maturation (Damerval et al., 2021). We suggest that the shape irregularities observed for *H. fulva* tepals indicates a level of responsive developmental flexibility that can result in structural complexity (Jiang and Moubayidin, 2022). Indeed, “symmetry breaking” is reported to be an adaptive strategy to adjust floral interactions with pollinators (Endress, 1999; Mora-Carrera et al., 2019).

This hypothesis is consistent with the observation that the stamens and stigma-styles of mature *H. fulva* flowers exhibit a sigmoidal curvature (**Figure 1**), which achieves different degrees of bilateral symmetry in the entire flower depending on the orientation of flowers with respect to the horizontal. This phenomenon can result in a widespread, weak or strong bilateral symmetry, as observed in many other angiosperm species (Citerne et al., 2010; Endress, 2012; Naghiloo, 2020). This type of bilateral symmetry is directly influenced by the position of the flower and may provide a precondition for the evolution of more elaborate bilateral symmetry (Endress, 2012; Naghiloo, 2020). In addition, symmetry may change during flower development, with the symmetry in early developmental stages differing from that in the mature flower (Damerval et al., 2021; Jiang and Moubayidin, 2022).

For example, Vincent and Coen (2004) report that in *Antirrhinum majus*, the early meristem shows bilateral symmetry. At sepal initiation, the bud is nearly radially symmetrical, but subsequently develops into and maintains bilateral symmetry. In the case of *H. fulva*, the sigmoidal curvature of the stamens and stigma commonly develops late in floral development (Endress, 2012). Thus, it is possible that in the early stages of *H. fulva* flower development, the stamens and stigma have not yet curved (i.e., the floral symmetry of *H. fulva* is radial) and only later assume varying degrees of bilateral or asymmetric morphology. Ontogenetic analyses are required to evaluate this proposition.

### 4.2 Comparison of inner and outer whorl tepals

A variety of metrics used in this study [i.e., FM, DM, *A*, *W*/*L*, RI, and ln(SI)] indicate that there are statistically significant differences in size and shape between the inner and outer whorls of *H. fulva* (**Figure 4**). The tepals in the inner whorl are larger, broader, and have a lower degree of deviation of tepal shape from a standard circle, and a greater degree of bilateral symmetry compared to the outer whorl. These trends are consistent with an incipient differentiation between sepals and petals reflecting different functionalities. For example, the tepals in the inner whorl may provide positional cues for pollinators, whereas the tepals of the outer whorl may provide protection during the development of stamens and carpels. Similar proposals have been presented (Citerne et al., 2010; Jiang and Moubayidin, 2022).

In addition, the 95% CIs of the scaling exponents of FM and DM vs. *A* for the outer whorl tepals include unity (**Figure 5**). In contrast, the lower bounds of the 95% CIs of the scaling exponents of FM vs. *A* and DM vs. *A* for the inner whorl tepals exceed unity (**Figure 5**), which is consistent with the phenomenon called “diminishing returns” (Niklas et al., 2007). These differences once again likely reflect different functionalities. For example, each tepal need only bear its own weight or that of neighboring tepals, as well as dynamic forces, such as wind (Gardiner et al., 2016). Wind pollination, a key mechanism in many plant species (Regal, 1982; Wang et al., 2019; Butcher et al., 2020), further contributes to the environmental pressures faced by tepals. The inner whorl tepals, which have a significantly larger area compared to the outer whorl (**Figure 4**), are closer to the stamens and stigma compared to the outer whorl tepals, and may provide positional cues for pollinators. Together, these factors may explain the “diminishing returns” phenomenon between mass and area for the inner whorl tepals.

### 4.3 Scaling relationships of mass vs. area for tepals and leaves

Classical botanical theory interprets floral parts as metameric homologues of foliage leaves, a concept of serial homology that emerged from the writings of J. W. von Goethe (Wolff, 1774; Goethe, 1790; Eyde, 1975; Bailey, 2008; Guo et al., 2022). The 95% CIs of the scaling exponents of FM and DM vs. *A* include unity for the outer whorl tepals of *H. fulva* (**Figure 5**), indicating that these relationships are isometric. In contrast, for the inner whorl tepals, the lower bounds of the 95% CIs of the scaling exponents of FM and DM vs. *A* exceed unity (**Figure 5**), indicating that increases in tepal area fail to keep pace with increases in tepal mass, consistent with the phenomenon called “diminishing returns” (Niklas et al., 2007). Similarly, increases in leaf lamina area fail to keep pace with increases in leaf mass, as evidenced by the lower bounds of the 95% CIs of the scaling exponents of LFM vs. LA and LDM vs. LA, which exceed unity (**Figure 6**). This may be due to the slender and elongated nature of the leaves of *H. fulva* (**Figure 1**), which not only bear more static weight from the upper parts of the plant but also withstand greater dynamic forces, such as wind, compared to flowers (Gardiner et al., 2016). Although the homology between floral parts and leaves has not been directly confirmed, our data can be interpreted to indicate that the leaves and perianth parts of *H. fulva* may have evolved distinct adaptive biomass allocation strategies, particularly in terms of mechanical traits.

## 5 Conclusions

This study provides additional insights into the floral symmetry of *H. fulva* and explores the scaling relationships of tepal mass vs. area and leaf mass vs. area. Significant differences in size, shape, and the scaling relationship between tepal mass and area for the inner and outer whorl tepals were detected, which can be interpreted to indicate that the adaptive functionalities of the inner and outer whorls differ. The scaling relationships between tepal mass and area, as well as leaf mass and area, reveal a finely tuned balance in resource allocation and mechanical performance. Perhaps more important, the data indicate that different metrics for measuring size and shape can yield what appear to be conflicting assessments of symmetry, which highlights the challenge of evaluating biological symmetries. Future studies are required to explore how floral symmetry and scaling relationships influence ecological adaptation and pollination efficiency in other plant species.

## Data Availability

The original contributions presented in the study are included in the article/**Supplementary Material**. Further inquiries can be directed to the corresponding authors.
